# Supplementary figures and images for: Use of virtual reality in the reduction of pain after the administration of vaccines among children in primary care centers in Central Catalonia: Randomized clinical trial
Source: PLoS One. 2025 May 7;20(5):e0322840. doi: 10.1371/journal.pone.0322840 (PMC12057881; doi:10.1371/journal.pone.0322840)

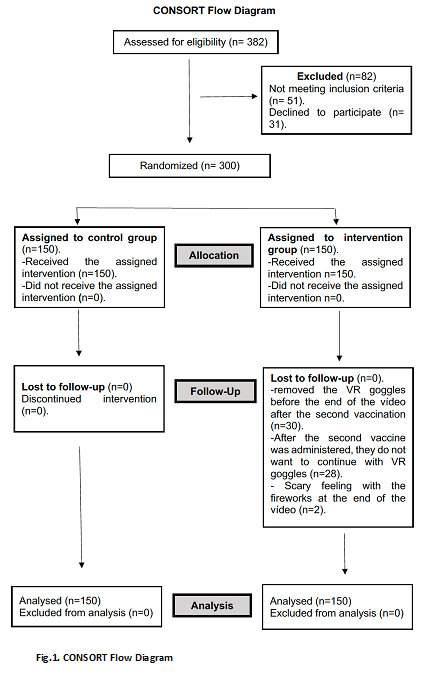

Supplement: S2 File — (TIF) [file pone.0322840.s002.tif]
