# Supplementary material for: Use of virtual reality in the reduction of pain after the administration of vaccines among children in primary care centers in Central Catalonia: Randomized clinical trial
Source: PLoS One. 2025 May 7;20(5):e0322840. doi: 10.1371/journal.pone.0322840 (PMC12057881; doi:10.1371/journal.pone.0322840)
Supplement: Attachment — Submitted filename: Response to Reviewers.docx [file pone.0322840.s004.docx]

Dear Editor,

We sincerely appreciate the time and effort invested in reviewing our manuscript and the constructive feedback provided. We have carefully considered all the comments and have made the necessary revisions accordingly. Below, we provide a point-by-point response to each of the concerns raised, detailing the modifications implemented in the revised manuscript.

**Keywords:** keywords have been reduced.

**Abstract:** they have been separated into sections as recommended and ending with the clinical trial registration number.

**Formatting**: the spaces between paragraphs have been reviewed and any excess have been corrected.

**Sample size justification / multicenter details / randomization and allocation / proportional allocation**:

The sample size calculation, the method used to obtain the sample, and the calculator employed are justified in the manuscript review. Additionally, we have explicitly explained the rationale behind selecting a standard deviation of 3. Furthermore, we have detailed the simple 1:1 randomization process applied to the population that met the inclusion criteria across the five centers. These clarifications have been included in the revised manuscript.

The manuscript clearly states that blinding was not feasible for either participants or healthcare personnel. This was specified in the initial version of the manuscript and has been further emphasized in the revised version for clarity.

**Statistical Analysis**:

For greater clarity, the medians of the variables have been provided in the revised manuscript. These additions have not influenced the previous results, ensuring consistency with the original findings.

**Outcome Assessment**: these have been specified in the initial paragraph of the description of each of the results and in the tables.

**Results and Discussion**:

The tables presenting the results for the variables pain and anxiety have been merged as recommended, allowing for a more structured discussion that follows the development of these key points. Additionally, the limitations of the study and conclusions, which were already included in the initial version, have now been explicitly specified as sub-sections for greater clarity.

**Reference**: we appreciate the recommendations, which have been taken into account when reviewing the bibliography.

**Please provide additional details regarding participant consent. In the ethics statement in the Methods and online submission information, please ensure that you have specified what type you obtained (for instance, written or verbal, and if verbal, how it was documented and witnessed). If your study included minors, state whether you obtained consent from parents or guardians. If the need for consent was waived by the ethics committee, please include this information.**

Written informed consent was obtained from the parents or legal guardians of all participants, as the study involved minors. This information was already included in the manuscript, and we have ensured that it is clearly stated in the Ethics Statement within the Methods section.

**Thank you for stating the following in the Competing Interests section:**

**"Mireia Cigarrán Mensa, cofounder and CEO of VRPharma Immersive Technologies SL, has an economic interest in the study. To mitigate any potential conflict of interest, VRPharma staff were not involved in data collection, statistical analysis, or the representation of results."**

**Please confirm that this does not alter your adherence to all PLOS ONE policies on sharing data and materials, by including the following statement: ""This does not alter our adherence to PLOS ONE policies on sharing data and materials.” (as detailed online in our guide for authors http://journals.plos.org/plosone/s/competing-interests). If there are restrictions on sharing of data and/or materials, please state these. Please note that we cannot proceed with consideration of your article until this information has been declared.**

We have included the requested statement in the manuscript: "This does not alter our adherence to PLOS ONE policies on sharing data and materials." There are no restrictions on sharing data or materials related to this study.

**In the online submission form, you indicated that "The datasets generated and/or analysed during the current study are not publicly available because our manuscript was based on confidential and sensitive health data. However, they are available from the corresponding author upon reasonable request."**

**All PLOS journals now require all data underlying the findings described in their manuscript to be freely available to other researchers, either 1. In a public repository, 2. Within the manuscript itself, or 3. Uploaded as supplementary information.**

**This policy applies to all data except where public deposition would breach compliance with the protocol approved by your research ethics board. If your data cannot be made publicly available for ethical or legal reasons (e.g., public availability would compromise patient privacy), please explain your reasons on resubmission and your exemption request will be escalated for approval**.

The datasets generated and analyzed during this study contain confidential and sensitive health data from pediatric participants. Publicly sharing these data would breach compliance with patient privacy regulations and the protocol approved by our ethics committee. According to the ethical approval obtained, the data cannot be made publicly available. However, they can be accessed from the corresponding author upon reasonable request, following appropriate ethical and legal safeguards to ensure participant confidentiality. We kindly request an exemption from the open data policy due to these ethical constraints.

**Reviewer #2: My Comments to authors.**

Dear Reviewer,

We sincerely appreciate your time and valuable comments, which have helped improve the clarity and quality of our manuscript. Below, we provide a detailed, point-by-point response to your suggestions and outline the modifications made accordingly.

**1. Why are these all keywords, which are about 13? It is good keywords to be 5-7.**

We have revised the keywords section and now include only 5 keywords to comply with the journal’s guidelines.

**2. Why are method and finding merged in the abstract part?**

We have restructured the abstract to clearly separate the methods and findings sections, ensuring clarity and adherence to standard formatting.

**3. Since this study is a randomized clinical trial, the registration number and name of the trial registry should be included at the end of the abstract. Why is it in the ethical considerations section?**

We have moved the trial registration number to the end of the abstract, as recommended, and removed it from the ethical considerations section.

**4. Remove the spaces between the paragraphs**

We have corrected the formatting by ensuring consistent paragraph spacing throughout the manuscript.

**5. The sample size calculation is not clear. How was sample size determined? How did you come up with 300 children? Of course you mentioned assumptions**

The sample size calculation, the method used to obtain the sample, and the calculator employed are now justified in the manuscript. Additionally, we have explicitly explained the rationale behind selecting a standard deviation of 3. The 1:1 randomization process applied to the population that met the inclusion criteria across the five centers has also been clarified.

**6. This is multicentre study, as you said (five primary care centers of the Catalan Institute of Health, in the territorial management of Central Catalonia – Súria, Callús, Valls de Torrouella, Cardona and Sant Frúitos de Bages-.) How many participant were selected from each primary care centers? Sampling procedure? Sampling technique?**

We have added details about the sampling procedure used in the Methods section. We have data about the recruitment in each primary care centre but we decided not to include it as there were no significant differences between intervention and control groups.

**7. Allocation concealment is good, but randomization? Is it individual randomization? Please clarify randomization.**

We have specified that individual randomization was used and provided additional details on the randomization procedure in the methodology.

**8. How were 39.3% 3 years old and 60.7% 6 years old being recruited? Is this a proportional allocation?**

The allocation was not proportional; the distribution of participants by age resulted from the natural recruitment process based on the availability of eligible children in each age group.

**9. What about blinding? Who was blinded after assignment to interventions? How was it impossible to blind?**

Blinding was not possible in this study because the children were aware of whether they were wearing the virtual reality glasses or not. As a result, both participants and healthcare personnel administering the vaccines were aware of the assigned intervention.

**10. You have measured the outcome variable two times (first and second vaccination times). This can be considered a repeated measure. So, why not consider other advanced statistical analyses like GLM, GEE, etc.?**

For greater clarity, we have provided medians of the variables, which have not influenced the previous results. While advanced statistical models were considered, we found that the current approach provided a clear and meaningful interpretation of the findings.

**11. Please include how and when outcome variables were assessed.**

We have provided additional details on the timing and methodology of the outcome assessments, specifying when pain, anxiety, and heart rate measurements were recorded.

**12. The results part is good**

Thank you.

**13. Please try to improve the beginning part of the discussion. First, you have to mention your finding, interpret the finding, and then compare with others**

We have restructured the Discussion section to begin with a clear statement of the main findings, followed by their interpretation and a comparison with previous literature, as recommended.

**14. Some references needs URL and accessed date, e.g ref no. 3**

We have reviewed and updated all references, ensuring that URLs and access dates are included where applicable.
