# Supplementary material for: Use of virtual reality in the reduction of pain after the administration of vaccines among children in primary care centers in Central Catalonia: Randomized clinical trial
Source: PLoS One. 2025 May 7;20(5):e0322840. doi: 10.1371/journal.pone.0322840 (PMC12057881; doi:10.1371/journal.pone.0322840)
Supplement: Attachment — Submitted filename: Response_to_Reviewers_auresp_2.docx [file pone.0322840.s005.docx]

**RESPONSE TO REVIEWER’S COMMENTS:**

**Reviewer #1: Tables 2 and 3: use decimal points instead of "comma". Be consistent with decimal numbers. The tables showed both 1 and 2 decimal numbers for percentages.**

Thank you for your feedback. We have made the requested changes to ensure consistency in decimal formatting. Specifically:

- Tables 1 and 2: We have replaced commas with decimal points for numerical values.
- Table 4: We have adjusted some results to maintain consistency in decimal formatting

**Journal guidelines:**

The abstract has been adjusted to 300 words taking into account the publication guidelines of the journal.
